# Comprehensive structural annotation of *Pichia pastoris* transcriptome and the response to various carbon sources using deep paired-end RNA sequencing

**DOI:** 10.1186/1471-2164-13-738

**Published:** 2012-12-31

**Authors:** Shuli Liang, Bin Wang, Li Pan, Yanrui Ye, Minghui He, Shuangyan Han, Suiping Zheng, Xiaoning Wang, Ying Lin

**Affiliations:** 1 School of Bioscience and Bioengineering, South China University of Technology, Guangzhou, Guangdong 510006, China; 2 School of life Science, General Hospital of PLA, Beijing 100853, China; 3 Beijing Genomics Institute at Shenzhen, Shenzhen 518000, China

**Keywords:** RNA-Seq, Transcriptome, *Pichia pastoris*, Methanol induction, Internal ribosome entry site (IRES), Translation initiation mechanism

## Abstract

**Background:**

The methylotrophic yeast *Pichia pastoris* is widely used as a bioengineering platform for producing industrial and biopharmaceutical proteins, studying protein expression and secretion mechanisms, and analyzing metabolite synthesis and peroxisome biogenesis. With the development of DNA microarray and mRNA sequence technology, the *P. pastoris* transcriptome has become a research hotspot due to its powerful capability to identify the transcript structures and gain insights into the transcriptional regulation model of cells under protein production conditions. The study of the *P. pastoris* transcriptome helps to annotate the *P. pastoris* transcript structures and provide useful information for further improvement of the production of recombinant proteins.

**Results:**

We used a massively parallel mRNA sequencing platform (RNA-Seq), based on next-generation sequencing technology, to map and quantify the dynamic transcriptome of *P. pastoris* at the genome scale under growth conditions with glycerol and methanol as substrates. The results describe the transcription landscape at the whole-genome level and provide annotated transcript structures, including untranslated regions (UTRs), alternative splicing (AS) events, novel transcripts, new exons, alternative upstream initiation codons (uATGs), and upstream open reading frames (uORFs). Internal ribosome entry sites (IRESes) were first identified within the UTRs of genes from *P. pastoris*, encoding kinases and the proteins involved in the control of growth. We also provide a transcriptional regulation model for *P. pastoris* grown on different carbon sources.

**Conclusions:**

We suggest that the IRES-dependent translation initiation mechanism also exists in *P. pastoris*. Retained introns (RIs) are determined as the main AS event and are produced predominantly by an intron definition (ID) mechanism. Our results describe the metabolic characteristics of *P. pastoris* with heterologous protein production under methanol induction and provide rich information for further in-depth studies of *P. pastoris* protein expression and secretion mechanisms.

## Background

The methylotrophic yeast *P. pastoris* is widely used as a heterologous expression platform for the industrial production of a series of valuable proteins due to its excellent characteristics, such as highly inducible gene expression, high-density cell growth, and high secretory capability [1]. The N-glycosylation pathway in *P. pastoris* has been reengineered to produce heterologous pharmaceutical proteins with human-like N-glycan structures, which may further improve the importance of *P. pastoris* in the biopharmaceutical industry [2-4]. *P. pastoris* is also used as a model organism to study the protein expression machinery, such as protein folding and secretion [5-9]. Apart from applications in protein expression, *P. pastoris* could also be used to study the biogenesis and degradation of the peroxisome [10].

Although the *P. pastoris* expression system has been investigated in numerous studies and has been commercially available for many years, there is still little physiological or genetic information available. A draft genome sequence of *P. pastoris* is now commercially available, but the strict obligation to keep the sequence information confidential has hampered the publication of relevant data [11]. Due to the lack of reported *P. pastoris* genome sequence and related DNA microarrays, alternative approaches such as heterologous hybridization of *P. pastoris* cDNA with *Saccharomyces cerevisiae* microarrays [12] and transcript analysis with the aid of affinity capture (TRAC) [5] have been exploited to study the *P. pastoris* transcriptome. The first DNA microarray for *P. pastoris* was produced using commercial sequence data (Integrated Genomics, Chicago, IL, USA), containing partial *P. pastoris* genes, and examined the unfolded protein response during protein production [6]. Recently, the genome sequences of three *P. pastoris* strains (GS115 [13], DSMZ70382 [14], CBS7435 [15]) have become publicly available. Transcriptomics, proteomics, and metabolic flux analysis data for *P. pastoris* will benefit from this now-public sequence information.

Transcriptomics is a favored approach for analyzing mRNA regulation patterns, providing snapshots of various physiological conditions or developmental stages. Recently, a massively parallel mRNA sequencing platform (RNA-Seq), based on next-generation sequencing technology, was used to map and quantify the dynamic transcriptome. Although RNA-Seq is a recent technology, and is still in active development, it has clear advantages over existing hybridization-based or tag sequence-based approaches for transcriptome analysis [16]. It offers key advantages, detecting known transcripts at single-nucleotide resolution, measuring gene expression levels over a larger dynamic range, discovering new transcripts within the intronic and intergenic regions, characterizing antisense transcription and RNA editing, and identifying alternative splicing events and gene fusion phenomena, as described in several reviews [16-18]. RNA-Seq data also show a high level of reproducibility, for both technical and biological replicates [19].

To date, RNA-Seq has been used successfully to define the transcription landscape on a genome-scale for over a dozen higher eukaryotic organisms, ranging from animals to plants [18]. *S. cerevisiae*[20-22], *Schizosaccharomyces pombe*[23], *Candida albicans*[24], *Aspergillus oryzae*[25], *Aspergillus flavus*[26], and several prokaryotes [27-29] have also been subjected to transcriptome analysis using the RNA-Seq technology.

On an industrial scale, the majority of *P. pastoris* processes described so far use glycerol as the substrate for fast growth to obtain high cell densities and methanol as the substrate and inducer for heterologous protein production. In this research, we sequenced *P. pastoris* poly(A)-enriched mRNA from growth conditions using glycerol or methanol as a substrate. We investigated the complex transcriptome of *P. pastoris* on a genome-scale using the RNA-Seq technology. Our results determined the transcription landscape on a whole *P. pastoris* genome scale (99.21%) and transcriptional level for the majority of *P. pastoris* annotated genes (4914 of the total of 5313 protein-encoding genes), defined 27 novel transcripts, four new exons, and untranslated regions (UTRs) for more than 900 genes. Alternative upstream initiation codons (uATGs) and upstream open reading frames (uORFs) in the 5’-UTRs of many genes were also identified. Internal ribosome entry sites (IRESes) were identified within the UTRs of the genes encoding kinases and proteins involved in growth control. Regarding AS events in the *P. pastoris* transcriptome, retained introns (RIs) was determined to be the main AS event and were produced predominantly by an intron definition (ID) mechanism. Based on the RNA-Seq data, we have depicted the differential gene expression of *P. pastoris* between growth conditions using glycerol and methanol as substrates and enriched the transcriptomic data available for *P. pastoris*.

## Results and discussion

### Summary of RNA-Seq data

To provide an analysis of the *P. pastoris* transcriptome at single base-pair resolution, cDNA libraries were constructed from poly (A)-enriched mRNA of *P. pastoris* chemostat cultures and analyzed using high-throughput Illumina sequencing. A paired-end sequencing strategy was used in RNA sequencing, in which the read length was augmented to 75 base pairs (bp). RNA samples were prepared from the chemostat cultures of *P. pastoris* GS115 transformed with different expression vectors bearing a lipase gene from *Rhizomucor miehei* (*RML*), including a secretory expression plasmid, a surface display expression plasmid, and a plasmid without the *RML* gene (control) (Additional file 1: Figure S1). When reaching the steady state, cell dry weights of *P. pastoris* strains were determined and samples were retained to isolate the RNA. The cell dry weight of CKM, SEM, SDEM_replicate1, SDEM_replicate2, SDEG_replicate1, and SDEG_replicate2 was 7.5, 7.3, 6.7, 6.9, 11.5 and 11.2 g/L, respectively.

**Figure 1:**
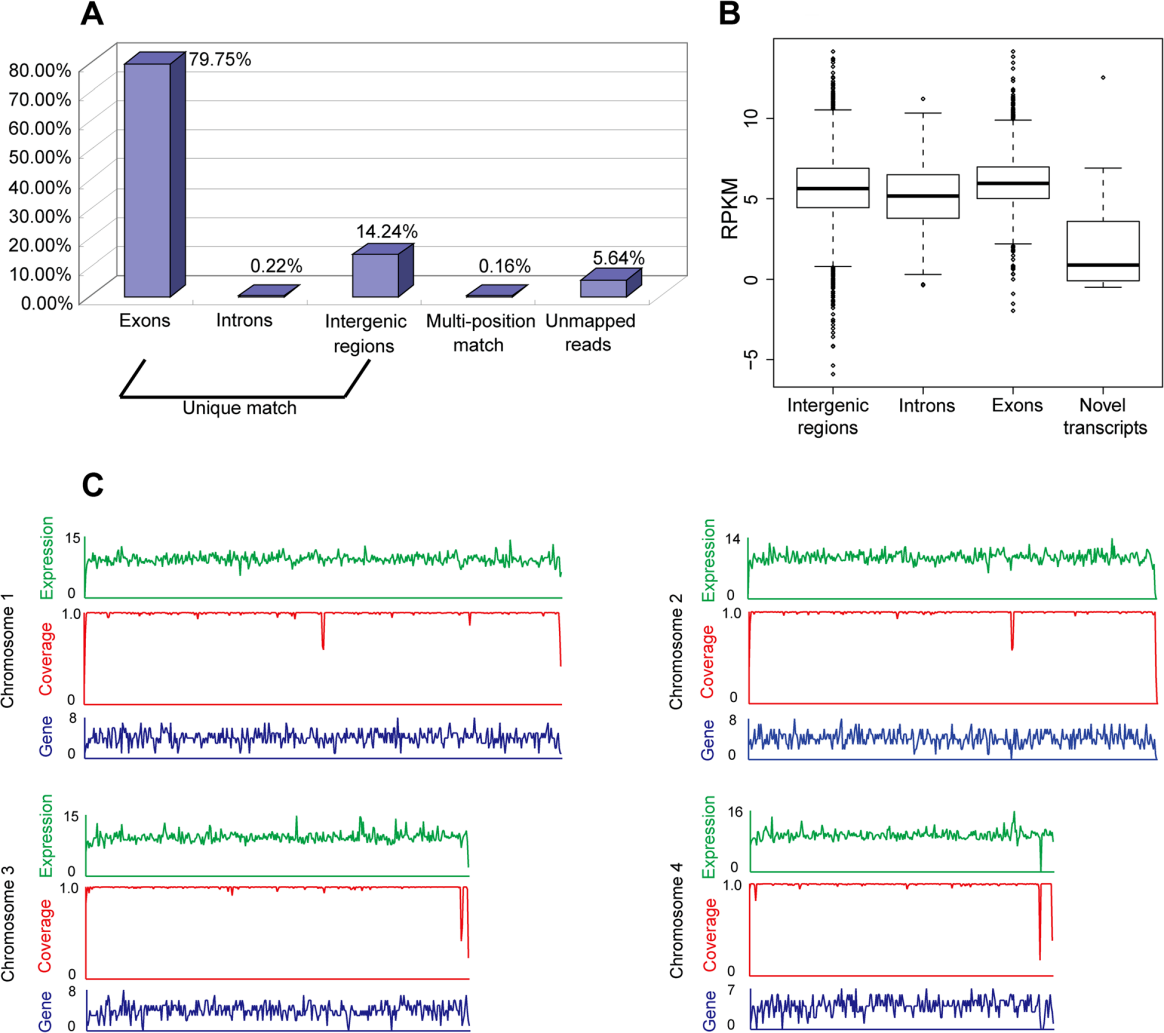
**Mapping summary of RNA-Seq reads and transcriptional distribution in *P. pastoris *genome. (A)** Statistics of RNA-Seq reads mapped in *P. pastoris* genome. Uniquely mapped reads include the reads located in exons, introns and intergenic regions. **(B)** Box-and-whisker plots of log_2_-transformed RPKM for the following regions: intergenic regions, introns, exons and novel transcripts. Diamonds represent data outside of the 5^th ^or 95^th ^percentile. **(C)** Expression profile denoted by log_2_-transformed reads count, depicting the transcriptionally active region (TAR) in the *P. pastoris* genome. “Coverage” depicts the percentage of the *P. pastoris* genome covered by RNA-Seq reads, and “Gene” depicts the number of BOGAS *P. pastoris* genes in a window size of 6 kilobases. The chromosome numbers are shown on the left.

In total, we obtained 152,711,406 reads within the samples cultivated with methanol as the substrate, representing ~1200 *P. pastoris* genomes (Additional file 2: Table S1), which gave enough information to accurately annotate the transcript structures. Of the total reads, 94.21% could be unambiguously mapped to the *P. pastoris* genome, of which 79.75% were mapped to known exons and 14.46% were located in intergenic and intronic regions (Figure 1A). The paired-end sequencing strategy effectively reduced the number of multi-position mapped reads to only 0.16% of the total reads (Figure 1A). This strategy, combined with long-read sequencing technology, improved the read matching from 85% (single-end reads) to 93% (paired-end reads) [30], and significantly increased the genome coverage, especially in the repeat regions [17]. The remaining 5.64% of the total reads were of poor quality (Figure 1A) and were discarded.

Extensive coverage of the complete *P. pastoris* genome was detected in the RNA-Seq data (Figure 1C). Of the genome, 99.21% was expressed as RNA-Seq reads (Additional file 2: Table S1). In the BioinformaticsGent Online Genome Annotation System (BOGAS), in total, 5313 protein-encoding genes were annotated in the 9.43Mbp genomic sequence of *P. pastoris* GS115 strain. Of all the annotated genes, > 93.5% were detected with >90% sequence coverage (Additional file 2: Table S2). However, using *S. pombe*, > 122 million reads with a length of 39 bp from six samples under different growth conditions, it was possible to detect transcription for virtually all of the annotated genes with >50% sequence coverage [23]. In this study, 152.7 million reads with a length of 75 bp provided a much more elaborate transcriptional status for *P. pastoris* annotated genes, detecting genes with lower expression levels, providing a powerful tool to dissect the detailed structure of the *P. pastoris* transcriptome at the single-base pair resolution.

To quantify gene expression levels by RNA-Seq data, the number of reads per kilobase of exon region per million mapped reads (RPKM) was calculated. The intronic and intergenic expression levels were only marginally lower than the expression level of exons (Figure 1B) and the mean expression level of novel transcripts was remarkably lower than these of intronic and intergenic regions. Overlapping transcriptional region in intergenic regions and antisense transcripts in intronic regions resulted in the higher expression level of intronic and intergenic regions. When an overlapping region occurs between two transcripts with opposite transcriptional orientation, its expression level was artificially elevated because reads mapping to both strands were all accounted. This becomes an issue especially in organisms with small genomes, where transcripts are densely packed. There were totally 1290 overlapping transcripts detected in the RNA-seq data (Additional file 2: Table S3). Boxplot analysis revealed that the expression level of overlapping transcripts was indeed much higher than their flanking regions (Additional file 1: Figure S2). Of the 5313 protein-coding genes in the *P. pastoris* genome database, 4970 were expressed as >1 RPKM with >90% sequence coverage in the RNA-Seq data (Additional file 2: Table S2). The control sample (CKM) without heterologous gene expression under conditions similar to *P. pastoris* natural growth conditions was used to depict its gene expression model (Additional file 1: Figure S3). Of all the annotated genes, 1204 genes were expressed greater than the 75^th^percentile (> 114.29 RPKM). The 6010 Go terms of the *P. pastoris* 2544 genes were downloaded from the BoinformaticsGent Online Genome Annotation Service (BOGAS) to perform Gene ontology (GO) analysis. Gene ontology (GO) analysis revealed that genes involved in the protein production system (structural constituent of the ribosome, proteasome, and translational elongation) and energy production system (ATP synthesis coupled proton transport, glycolysis, and mitochondrion) were specifically enriched in this highly expressed gene group (false discovery rate (FDR) <0.05). For expression levels ranging from the 25^th ^to 75^th ^percentiles (33.13-114.29 RPKM), 2407 genes were detected, where no highly enriched gene group was identified, according to GO functional enrichment analysis. The genes enriched in nucleic acid metabolic processes were expressed lower than the 25^th ^percentile. That is, the RNA-Seq data and related GO functional enrichment analysis clearly demonstrated that the expression levels of the genes involved in protein production and energy metabolism were high while that of the genes involved in nucleotide metabolism and genetic message transfer were low under CKM growth conditions, conforming to the physiological and metabolic status of *P. pastoris* under methanol-induced conditions.

### Novel annotation of *P. pastoris* transcriptome using RNA-Seq data

#### UTR annotation for *P. pastoris* genes

**Figure 2:**
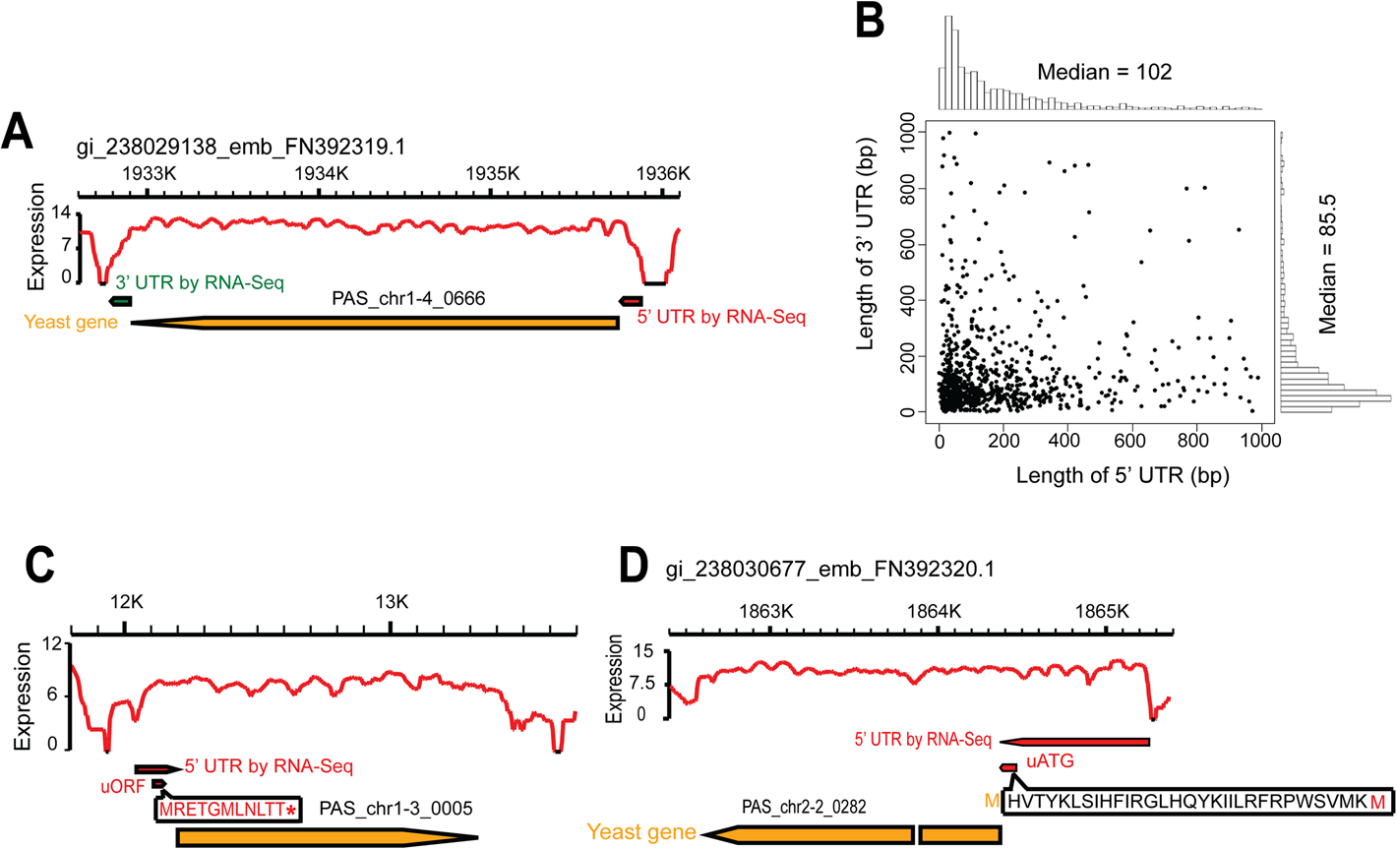
**Transcript structure annotation of *P. pastoris *by RNA-Seq data. (A)** UTR redefinition of the gene PAS_chr1-4_0666. **(B)** Length distribution of 5’ and 3’ UTRs of all annotated *P. pastoris* genes. **(C)** Annotated uORF in the 5’ UTR of gene PAS_chr1-3_0005. **(D)** Annotated uATG in the 5’ UTR of gene PAS_chr2-2_0282.

By searching for a sharp reduction in the expression signal at both ends of the annotated genes, we identified the 5’- and 3’-boundaries of the *P. pastoris* genes. The 5’-UTRs of 914 genes and 3’-UTRs of 924 genes were defined (Figure 2A and Additional file 2: Table S4). The median lengths of the 5’- and 3’-UTRs were 102 bp and 85.5 bp, respectively (Figure 2B), shorter than those of *S. pombe* (152 bp and 169 bp) [23]. There were 15 genes containing “ATG” codons upstream of their annotated start codon (uATG) in their 5’-UTRs, which may represent true initiation sites (Figure 2D, Additional file 2: Table S5). In many eukaryotes, 5’-UTRs contain upstream open reading frames (uORFs), which can regulate protein production and mRNA degradation [21]. Our data also predicted uORFs for 291 genes (5.5% of the total annotated genes; Figure 2C and Additional file 2: Table S6).

#### IRESes in the 5’-UTRs of *P. pastoris* genes

Translation initiation in the vast majority of cellular mRNAs is mediated by a cap-dependent mechanism. However, under many cellular conditions (mitosis, apoptosis, cellular stresses of hypoxia and heat shock, and viral infection) when cap-dependent translation initiation is compromised, protein synthesis is mediated via an alternative initiation pathway, the internal ribosome entry site (IRES) dependent translation initiation [31]. IRES elements have been found in mRNA from viruses, mammals, vertebrates, and yeasts [32]. However, IRES elements have not previously been demonstrated in *P. pastoris*, a widely used protein expression platform, because of a lack of UTR information. Within the database of published IRESes (IRESdb), cellular mRNAs-encoded IRESes are classified according to the gene product function. The IRES-containing mRNAs are always transcripted by the genes encoding transcription factors, translation factors, chaperones, kinases, and proteins involved in growth control [32,33]. Of the 5’-UTRs from 914 genes annotated in the present study, the 5’-UTRs of the protein kinase PAS_chr3_0850 (*GCN2*) and PAS_chr2-1_0459 (*KOG1*), involved in growth control, were selected and monitored for IRES activity.

**Figure 3:**
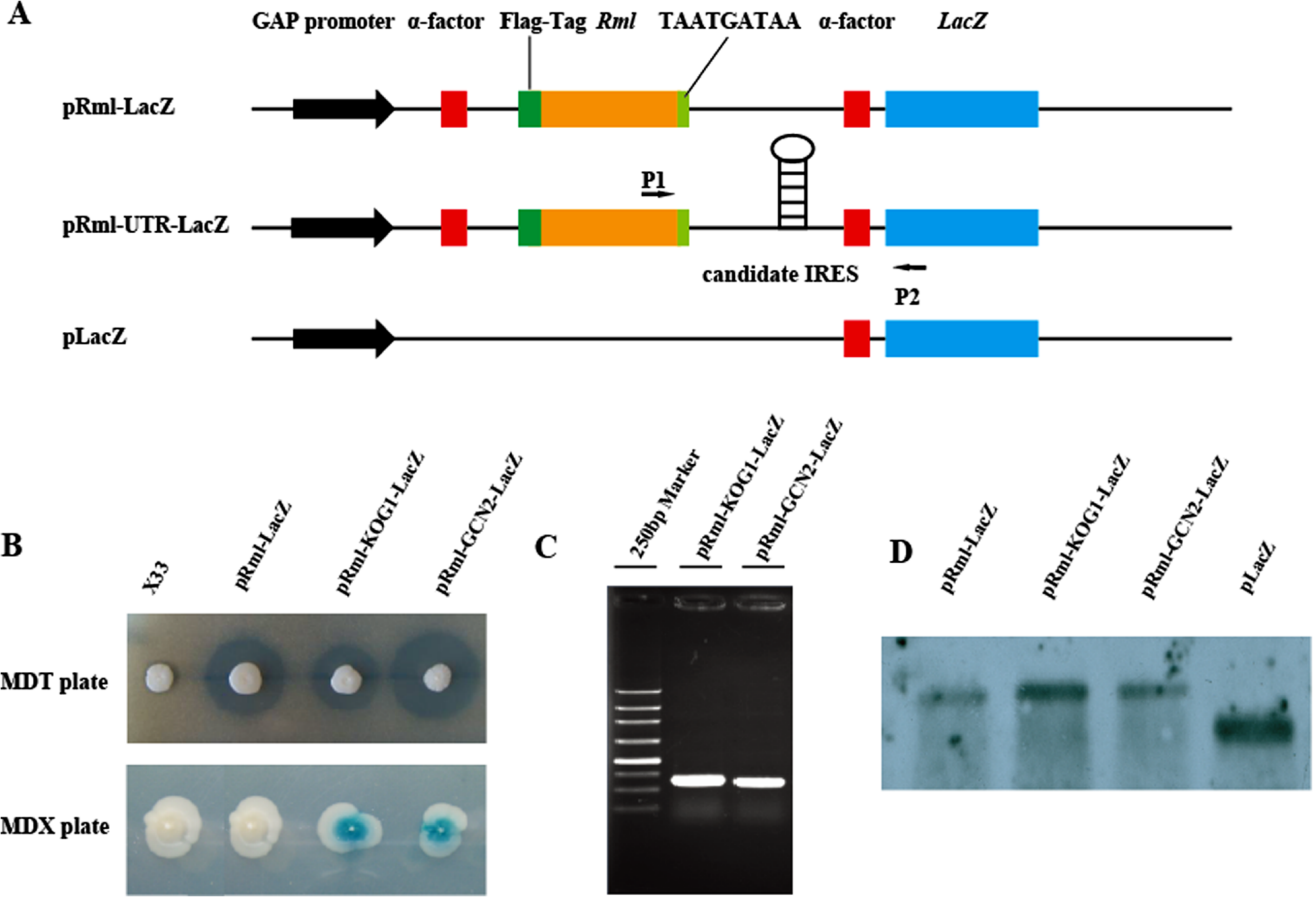
**IRES activity identification of 5’UTRs of *KOG1 *and *GCN2 *mRNA detected by RNA-Seq. (A)** Construction of vectors. The bicistronic vector pRml-LacZ containing the *RML* gene and the *LacZ* gene used as the negative control. Candidate IRESes were inserted between the *RML* gene and the *LacZ* gene, generating the pRml-UTR-LacZ. The expression of *LacZ* ORF in another control plasmid pLacZ was controlled by the GAP promoter. The arrows represent the primers used in PCR identification. **(B)** Detection of RML and β-galactosidase on MDT plate and MDX plate, respectively. **(C)** Identification of the integrity of bicistronic mRNAs with primer P1/P2. **(D)** Northern blot analysis of the transcripts from total RNA isolated from *P. pastoris* X33/pRml-LacZ, X33/pRml-GCN2-LacZ, X33/pRml-KOG1-LacZ, and X33/pLacZ.

To examine whether the chosen UTRs mediated IRES-dependent translation initiation, an upstream *R. miehei* lipase (*RML*) gene and a downstream *Escherichia coli* BL21 β-galactosidase gene (*LacZ*) were separated by the selected UTRs (Figure 3A). The recombinant *P. pastoris* strains were cultivated on MDT plates to detect expression of the *RML* gene and on MDX plates to monitor the synthesis of β-galactosidase. As shown in Figure 3B, the 5’-UTRs of *KOG1* and *GCN2* mediated the expression of the second cistron. In addition, PCR identification, with the corresponding cDNA as a template, proved the integrity of the bicistronic mRNAs and eliminated the possibility of RNA splicing events (Figure 3C). Furthermore, we performed Northern blot analysis to exclude cryptic promoter activity somewhere upstream of the *LacZ* coding sequence (Figure 3D). As shown in Figure 3A, we constructed a control plasmid pLacZ, in which the expression of LacZ ORF was controlled by the GAP promoter. For each *P. pastoris* strain, a single signal was detectable in the corresponding immunoblot. The size of the dual reporter construct containing an upstream *R. miehei* lipase (*RML*) and a downstream β-galactosidase gene (*LacZ*) was longer than the *LacZ* ORF, indicating that there was no cryptic promoter activity somewhere upstream of the *LacZ* coding sequence. These results suggested that the 5’-UTRs of *KOG1* and *GCN2* contained functional internal ribosomal entry sites. Due to the powerful ability for identifying UTR sequences of genes, next-generation massively parallel mRNA sequencing (RNA-Seq) will undoubtedly promote the study of IRES-dependent translation initiation mechanisms in *P. pastoris*.

#### Novel transcripts and exons in the *P. pastoris* genome

**Figure 4:**
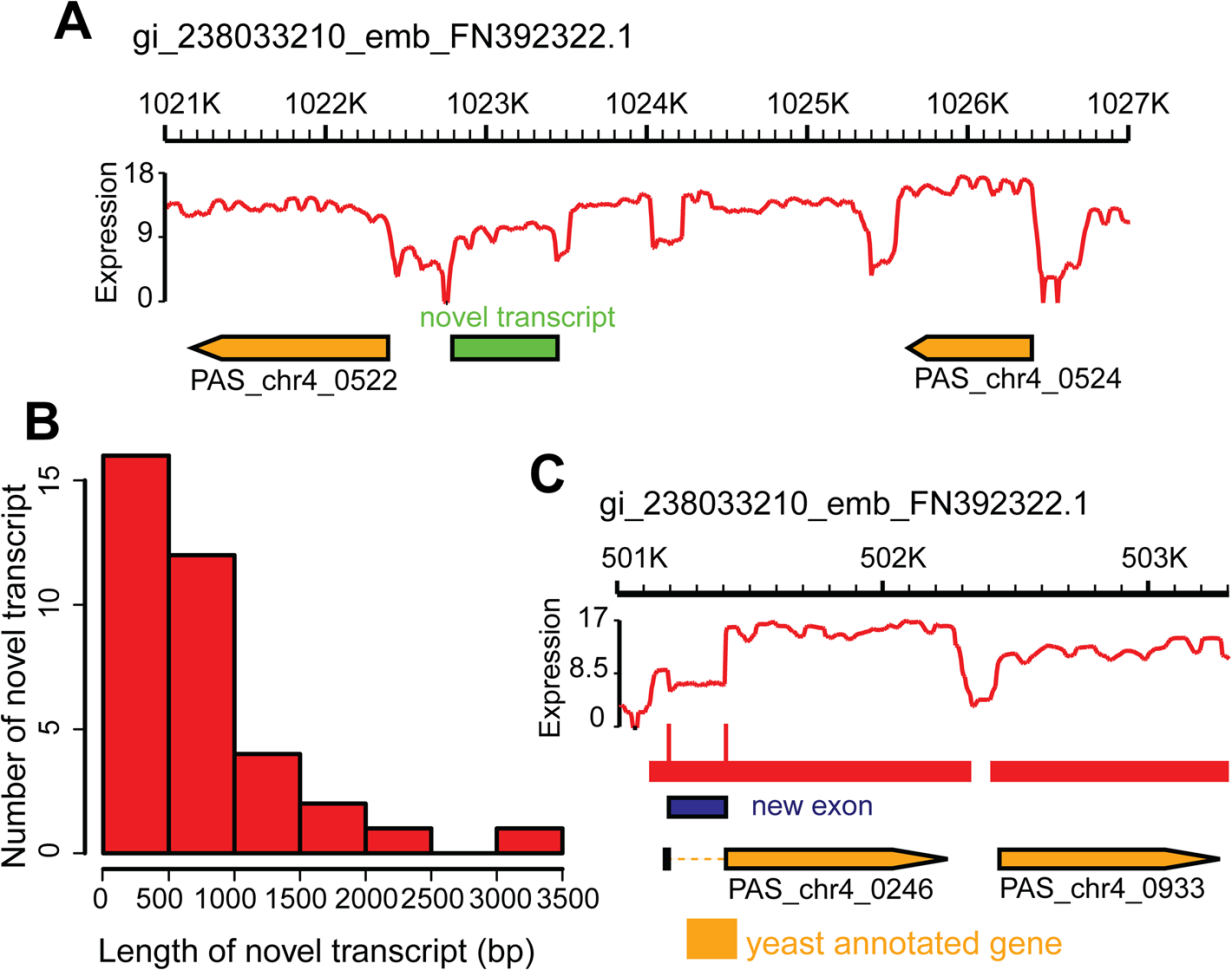
**Novel transcripts and new exons detected in the *P. pastoris *transciptome by RNA-Seq data. (A)** A novel transcript with considerable expression level in contig FN392322 (GI number: 238033210). **(B)** Length distribution of all detected novel transcripts. **(C)** A new exon detected in gene PAS_chr4_0246.

Based on the RNA-Seq data, the intergenic and intronic regions of the *P. pastoris* genome contained fragments with considerable expression levels (Figure 1B). About 14.24% of the total reads uniquely mapped to the intergenic regions (Figure 1A). Thus, we searched reads covering the intergenic regions for the potential novel transcripts by identifying stretches of 150 bp or longer with expression levels higher than the surrounding regions. According to these limitations, 27 novel transcripts were detected, with an average length of 1318 bp, containing potential protein-encoding ORFs (Figure 4A and Additional file 2: Table S7). For the intronic regions, although only 0.22% of total reads fell within these genomic areas (Figure 1A), the average expression level reached about 5 RPKM (Figure 1B). We analyzed these expressed introns and identified four new candidate exons in four known genes (Figure 4C and Additional file 2: Table S8). Two novel transcripts and one candidate exon were further confirmed by RT-PCR (Additional file 1: Figure S4). It is worth mentioning that antisense transcription, convergent genes and overlapping genes may interrupt the annotation of the *P. pastoris* transcript structures, although the strand specificity of a new exon (new exon in gene chr4_0246, coordinates 501191-501406) was validated by a tagged RT-PCR method (data no shown).

### Cataloging of Alternative Splicing in *P. pastoris* under different growth conditions

**Figure 5:**
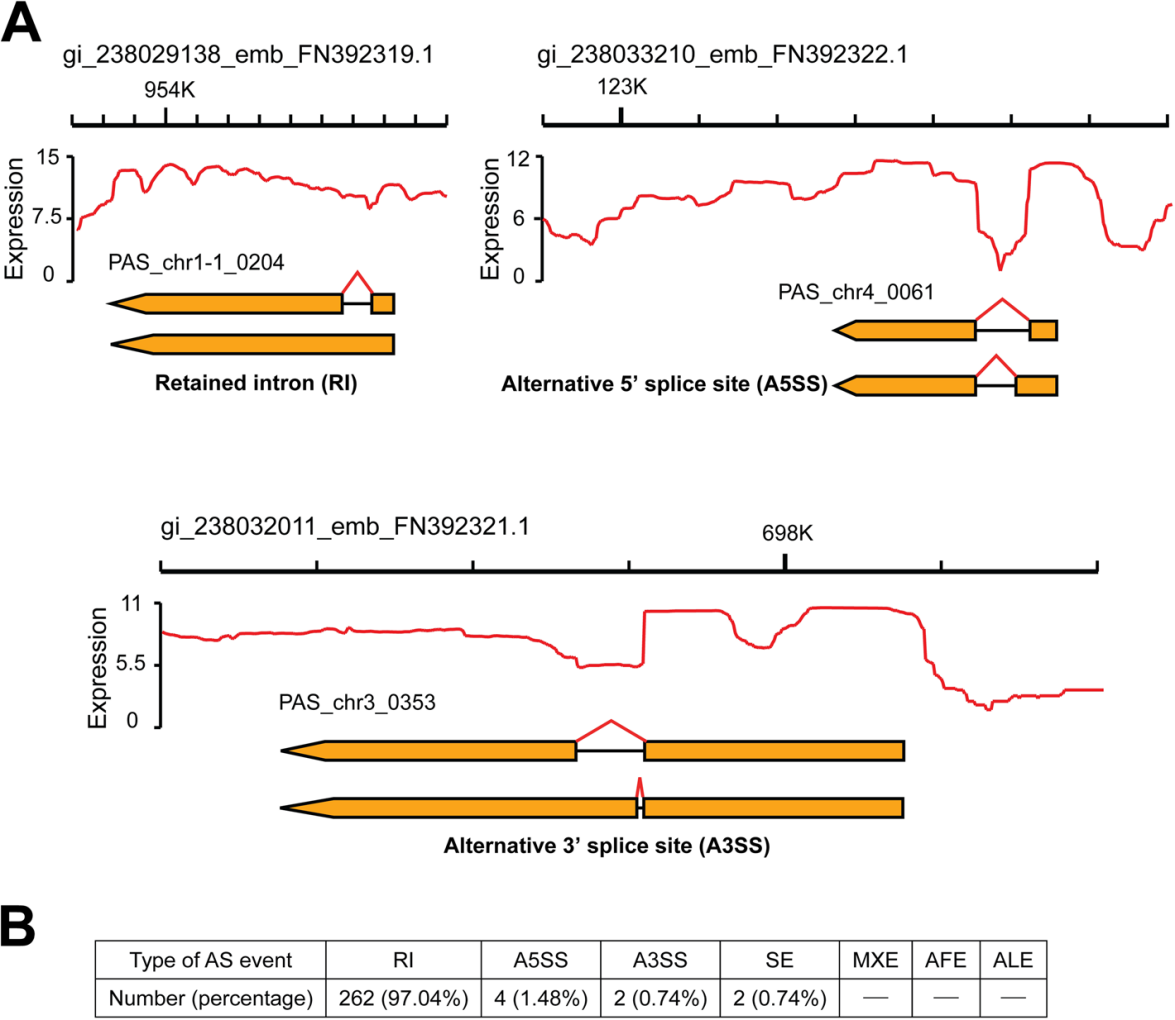
**AS events in *P. pastoris*. (A)** Examples of the main AS types in *P. pastoris*, including retained intron (RI), alternative 5’ splice site (A5SS) and alternative 3’ splice site (A3SS). The ordinate “Expression” represents the coverage depth of each genome location. **(B)** Statistics of AS events in *P. pastoris*.

Of all the *P. pastoris* genes, 11.91% (633 genes) contained two or more exons [13]. The average number of introns per gene was generally low, similar with *S.cerevisiae*[34]*.* Genome-wide detection of *P. pastoris* alternative splicing (AS) events was conducted in multi-exon genes by performing computational analyses to determine known and putative splicing junctions and to identify alternative splicing sites using stringent criteria (see Materials and Methods). Of the seven major AS types, only the retained intron (RI), alternative 5’-splice sites (A5SS), alternative 3’-splice sites (A3SS), and skipped exons (SE) were detected in *P. pastoris* (Figure 5A). In total, 254 (4.78%) *P. pastoris* genes underwent AS with a total of 270 AS events (Additional file 2: Table S9), which was the highest AS frequency in yeast. In contrast, only four AS events were detected in *S. cerevisiae *[22] under vegetative growth with heat shock and there was no evidence for the existence of AS events in *S. pombe* using the RNA-Seq technology [23]. Additionally, 8.55% of filamentous *A. oryzae* genes underwent AS events, detected by deep sampling of its transcriptome [25]. Previously it has been reported that the number and variety of introns and splice sites may be coupled with the phenomena of AS events [35], which was apparently applicable in *S. cerevisiae* and *A. oryzae*. In *S. cerevisiae*, 5% of all genes are spliced [36]. In *A. oryzae*, 76.98% of the total genes contain at least one intron [25]. However, there are sometimes exceptions. For example, numerous intron-containing genes (45.4% of all genes) do not undergo any AS events in *S. pombe*[23]. It is noteworthy that alternative splicing seldom yields a functional protein. Only the intron retention events of PAS_chr1-3_0077, PAS_chr2-1_0595 and PAS_chr3_1114 could produce functional proteins.

We performed GO functional enrichment analysis of *P. pastoris* alternatively spliced genes. Of all the alternatively spliced genes, 135 (53.36%) were annotated by GO enrichment analysis via Fisher’s exact test (FDR < 0.05). Regarding molecular function, genes producing proteins with ion transmembrane transporter activity and ion-transporting ATPase activity have a high frequency of AS events. Cellular component analysis revealed that genes encoding macromolecular complexes and protein complexes were specifically enriched in AS events. Biological process analysis showed that AS events occurred mainly in the processes of macromolecular complex assembly and cellular component organization.

RI is the dominant AS variant in fungi with great statistical significance (p < 1e-10 by Fisher’s exact test) [35]. Consistent with this, RI was detected as the main AS event in our *P. pastoris* RNA-Seq data (Figure 5B), accounting for 97.04% of all AS isoforms. In our study, RNA samples were subjected to DNase treatment to eliminate any possible contamination with genomic DNA. Therefore, RI events in our data should not be interfered with by intronic genome sequences. RI events were further validated by qPCR. The results illustrated that the retained intron of the PAS_chr1-1_0445 gene exhibited a transcriptional level equivalent to its annotated exon while the canonical intron had a relatively low transcriptional level (Additional file 1: Figure S5). AS events can be divided into four categories: RI, cassette exon (CE, including SE, alternative first exons (AFE), alternative last exons (ALE), and mutually exclusive exons (MXE)), A5SS, and A3SS. The ratio CE/(RI + CE) is usually used to summarize the pattern of the splice variants [35]. This ratio in *P. pastor*is was only 0.76% (Figure 5B), comparable to that of five formerly investigated fungi [35]. Further, it has been suggested that RI was the main AS type in *P. pastoris*. It has been reported that ID, as one of the splice site recognition mechanisms in eukaryotes, was prone to produce RI variants and the retained introns were usually shorter than the constitutive introns [35]. The average length of the *P. pastoris* retained introns was 89 bp, shorter than the canonical introns (99 bp) in *P. pastoris*. Thus, *P. pastoris* may perform splice site recognition predominantly by the ID mechanism.

### Analysis of differentially expressed genes in *P. pastoris* cultivated with glycerol and methanol

**Figure 6:**
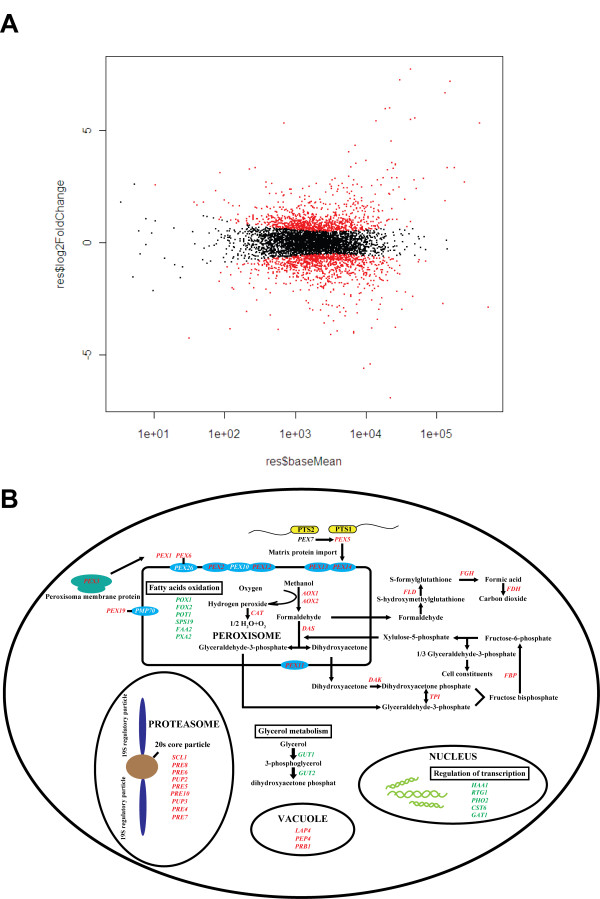
**Analysis of *P. pastoris *differential gene expression between SDEM and SDEG culture. (A)** Differential gene expression between *P. pastoris* SDEM and SDEG culture, analyzed by the ‘R’ (DESeq) software. **(B)** Metabolic pathway analysis of differentially expressed genes between SDEM and SDEG culture. Genes denoted by red font are up-regulated in SDEM condition compared to SDEG condition. Genes denoted by green font are down-regulated.

RNA-Seq technology, with its digital gene expression data, has been explored as a quantitative method to analyze differential gene expression between different physiological states of a given organism. In the industrial production of heterologous proteins, *P. pastoris* processes typically use glycerol as a substrate for cell growth and methanol as the substrate and inducer for heterologous protein production. Here, we detected globally differential expressed genes in *P. pastoris* between the SDEM culture and the SDEG culture, based on RNA-Seq data using the ‘R’ (DESeq) software. In order to rule out the influence on the gene expression due to copy number, we measured the copy number of AOX1 promoter of the *P. pastoris* strains and found that each strain was transformed with a single copy control vector or the expression vector (data not shown). To reduce conflicting expression variance in biological replicates, poly (A)-enriched mRNA for constructing RNA-Seq cDNA libraries were derived from chemostat cultures of *P. pastoris*. The two biological replicates of *P. pastoris* SDEM and SDEG cultures were in close agreement, with a 0.99 Pearson correlation coefficient. Differentially expressed genes were identified based on the negative binomial distribution with variance and mean linked by local regression [37]. In total, 1885 genes were differentially expressed under SDEM culture compared with SDEG culture; 940 were up-regulated and 945 were down-regulated (padj < 0.01, Additional file 2: Table S10 and Figure 6A).

Compared with the SDEG culture, in the SDEM culture, GO analysis and KEGG metabolic pathway analysis revealed that up-regulated genes were specifically located in proteasome, autophagy, phagosome, thiamine metabolism, N-glycan biosynthesis, methane metabolism, protein processing in the endoplasmic reticulum, and protein export pathways (Additional file 1: Figure S6). Genes involved in the biosynthesis of unsaturated fatty acids and the citrate cycle were down-regulated (Figure 6B and Additional file 2: Table S10). These results described the metabolic characteristics of heterologous protein production of *P. pastoris* with methanol induction. Carbon shift from glycerol to methanol is an important process in heterologous protein expression. The genes involved in the methanol utilization pathway were markedly up-regulated under SDEM conditions, similar to the adaptation of *Hansenula polymorpha* to methanol [38]. Alcohol oxidases (*AOX*, including *AOX1* and *AOX2*) were up-regulated by 39.8-fold and 11.3-fold, respectively; they catalyze the methanol to formaldehyde and hydrogen peroxide reaction as the first step of the methanol utilization pathway. Other related genes, such as catalase (*CAT*), formaldehyde dehydrogenase (*FLD*), and S-formyl glutathione hydrolase (*FGH*), were also highly expressed (see supplementary data). For glycerol metabolism, *P. pastoris* homologs of *S. cerevisiae* putative passive glycerol channel (*YFL054C*) and the active glycerol importer (*STL1*) decreased significantly at the transcriptional level after the carbon shift from glycerol to methanol. Cytosolic glycerol kinase (*GUT1*) was also down-regulated because of the lack of glycerol supply.

Another significantly up-regulated gene group was vitamin metabolism. As an important vitamin, thiamine can be synthesized from hydroxylmethylpyrimidine pyrophosphate and hydroxyethylthiazole phosphate by TMP synthase. All thiamine-regulated genes in *P. pastoris* were induced on the carbon shift from glycerol to methanol (Additional file 2: Table S10), which was consistent with the circumstances in the batch fermentation at pH5 or pH3 [12]. The up-regulation of these genes may be due to the activation of TPP riboswitches [39].

*P. pastoris* needs many more peroxisomes to resist the toxicity of methanol when methanol is the sole carbon source. In the SDEM culture, almost all of the genes controlling the development and the function of the peroxisome were up-regulated compared with the SDEG culture (Additional file 2: Table S10). Peroxisome proteins are encoded by nuclear genes, synthesized in the cytoplasm, and then imported into peroxisomes. Two peroxisomal targeting signal genes (*PTS1* and *PTS2*) are essential for sorting proteins to this organelle. *PEX5* and *PEX7* encode receptors for *PTS1* and *PTS2*, respectively [40]. The expression level of *PEX5* was much higher than that of *PEX7*, consistent with only a limited number of peroxisomal matrix proteins containing the *PTS2* receptor [40]. Both *PEX1* and *PEX6*, which act in the terminal step of the peroxisomal matrix protein import [41] and participate in the recycling of peroxisomal signal receptors, were up-regulated, indicating that the requirement for importing matrix proteins to peroxisomes was strong. The most up-regulated peroxisome gene was *PEX11* (11.96-fold), which is implicated in regulating peroxisome proliferation. The up-regulation of *PEX11* was consistent with previous findings in baker’s yeast [42] and *H. polymorpha*[38] cultivated with peroxisome-inducing carbon sources. It is thought that *PEX11* also participates in the β-oxidation of fatty acids [43]. *PEX11*, together with the genes related to β-oxidation of fatty acids, is induced in *H. polymorpha* by the carbon shift from glucose to methanol [38], but their expression levels are down-regulated in *P. pastoris* under hypoxic conditions [8]. Surprisingly, *PEX11* was induced while the genes related to β-oxidation of fatty acids (*POX1, FOX2, POT1, SPS19, FAA2, PXA2*) were down-regulated in our RNA-Seq data under methanol conditions. The reason for this difference remains unclear and needs further investigation.

In addition to glycerol metabolism, transcriptional regulation was the main biological process enriched in the down-regulated genes (>1.5-fold) when methanol was the sole carbon source. Down-regulation of genes encoding transcription factors (*RTG1*, *PHO2*, *CST6*, *HAA1*, and* GAT1*) was detected. It has been reported that the lower specific growth rate resulted in the down-regulation of genes related to ribosomal synthesis when the carbon source is changed from glycerol to methanol in recombinant *P. pastoris*[12]. Analogously, reduced transcriptional regulation would be due to the lower growth rate under methanol conditions in *H. polymorpha*[38]. A lower growth rate could not explain the reduced transcriptional regulation in this study because of the chemostat culture, suggesting that *P. pastoris* may be prone to reduce the transcription of some less important genes to supply enough energy and substrates for expression of the heterologous gene (here, *RML*).

## Conclusions

With high resolution and unprecedented throughput (~1200 *P. pastoris* genomes), the global transcriptome of *P. pastoris* was accurately annotated under the cells growth with glycerol as the substrate and the protein expression using methanol as an inducer and substrate. Internal ribosome entry sites were identified in the 5’-UTRs of *KOG1* and *GCN2,* indicating that the IRES-dependent translation initiation also exists in *P. pastoris*. Differential expression analysis provided rich information for further in-depth studies of the translation initiation mechanism, protein expression and secretion mechanisms in *P. pastoris*. These results will be very useful in the further bioprocess engineering and optimization of induced conditions of *P. pastoris.*

## Materials and methods

### Strains and vectors

The strains used for RNA-Seq are summarized in Table 1. *P. pastoris* GS115 (Invitrogen, USA), a histidine auxotrophic strain, was used as the host strain for the construction of recombinant strains. The secretory expression plasmid pPIC9K-Rml was constructed by inserting the coding sequence of the *R. miehei* lipase gene (*RML*) into the vector pPIC9K (Invitrogen, USA). Similarly, the surface display vector for the *RML* gene (pPIC9K-KNS-Rml) was obtained by inserting the fusion gene *RML-*α-agglutinin into the plasmid pPIC9K. The secretion signal peptide α-factor of pPIC9K and α-agglutinin were used as the secretion signal and the anchor protein for the cell surface display of *RML* protein in *P. pastoris.* Additionally, strain GS115/PK, which contains the vector pPIC9K without heterologous genes, was used as a control. Each *P. pastoris* strain was integrated with a single copy number of the respective plasmid.

**Table 1:** Strains and plasmids used for RNA-Seq

**Strains**	**Plasmids**	**Carbon resource**	**Abbreviation of samples**	**Short descriptions**
GS115/PK	pPIC9K	Methanol	CKM	Non-expression of heterlogous protein
GS115/Rml	pPIC9K-Rml	Methanol	SEM	Secretory expression of *RML* gene
GS115/KNS-Rml	pPIC9K- KNS-Rml	Methanol	SDEM	Surface display expression of *RML* gene
		Glycerol	SDEG	

### Chemostat cultivation

Chemostat cultivation was used throughout this study, providing constant environmental conditions to ensure that the yeast cultures for transcriptome analysis would be reproducible, reliable, and biologically homogeneous. To obtain seed cultures for chemostat cultivation, single colonies on fresh YPD plates (10 g/L yeast extract, 20 g/L peptone, 20 g/L glucose, 20 g/L agar) were inoculated directly into 250 mL shake flasks containing 100 mL YPD liquid culture medium. After cultivation at 28°C for approximately 24 h with agitation (250 rpm), seed cultures were used to inoculate the starting volume (1 L) of the bioreactor to an optical density (OD_600_) of 0.5. The chemostat cultivations, consisting of batch cell growth (batch) and continuous culture with glycerol or methanol feeding, were performed in a 2.0 L working volume bioreactor under computer-based process control.

Batch cultivations were performed in basal salt medium, containing (per L): 26.7 mL H_3_PO_4_ (85%), 0.93 g CaSO_4_, 18.2 g K_2_SO_4_, 14.9 g MgSO_4_·7H_2_O, 4.13 g KOH, 40.0 g glycerol, 0.5 mL antifoam, and 4.35 mL trace salt stock solution. Trace salt stock solution contained (per L): 6.0 g CuSO_4_·5H_2_O, 0.08 g NaI, 3.0 g MnSO_4_·H_2_O, 0.2 g Na_2_MoO_4_·2H_2_O, 0.02 g H_3_BO_3_, 0.5 g CoCl_2_, 20.0 g ZnCl_2_, 65.0 g FeSO_4_·7H_2_O, 0.2 g biotin, and 5.0 mL of H_2_SO_4_. The cultivation temperature was 28°C with a pH of 5.0, controlled with 25% ammonium hydroxide. The dissolved oxygen concentration was maintained above 20% saturation by altering the stirrer speed between 200 and 1200 rpm, while the airflow was kept constant at 2.0 vvm.

After approximately 24 h, the batch finished and the continuous culture started at a dilution rate of D = 0.04 h^-1^. The chemostat medium contained (per L): 13.4 mL H_3_PO_4_ (85%), 0.47 g CaSO_4_, 9.1 g K_2_SO_4_, 7.5 g MgSO_4_·7H_2_O, 2.07 g KOH, 20.0 g methanol or glycerol, 0.5 mL antifoam, and 4.35 mL trace salt stock solution. The continuous culture was performed for at least five resident times to reach steady-state conditions.

### RNA sequencing

For Illumina sequencing, total RNA was extracted from each sample using the hot acidic phenol method and then treated with RNase-free DNase I (TaKaRa) for 45 min according to the manufacturer’s protocol. The integrity of the total RNA was analyzed using an Agilent Technologies 2100 Bioanalyzer, and all six samples were shown to have an RNA Integrity Number (RIN) value > 8. The poly(A)-enriched mRNA was purified using Sera-mag Magnetic Oligo(dT) Beads (Illumina) from 20 mg total RNA method described by Wang et al [25]. The cDNA library products were sequenced using the 1 G Illumina Genome Analyzer at the Beijing Genomics Institute, Shenzhen. Two biological replicates of SDEM and SDEG were analyzed. The raw sequencing data are deposited in SRA [44] at NCBI with accession number SRA048068.

### Read mapping in the reference genome and genes

The *P. pastoris* GS115 genome and gene information were downloaded from the *P. pastoris* genome database [45]. After removing the reads containing sequencing adapters and reads of low quality (reads containing Ns > 4), the remaining reads were aligned to the *P. pastoris* genome using SOAP2 [46], allowing up to 5-base mismatches. The reads that failed to be mapped were progressively trimmed off one base at a time from the 3’-end and mapped to the genome again until a match was observed (unless the read had been trimmed to less than 40 bases). For paired-end reads, the insert between paired reads was set from 1 base to 10 kilobases, allowing them to span introns of varying sizes in the genome. The same strategy was used for aligning paired-end reads to the non-redundant gene, except the insert was changed from 1 base to 1 kilobases.

### Normalized gene expression using RNA-Seq

The gene expression level was normalized by the number of reads per kilobase of exon region per million mapped reads (RPKM) [47]. The cutoff value for determining gene transcriptional activity was determined based on a 95% confidence interval for all RPKM values of each gene. That is to say, the genes with an expression level in the lowest 5% were discarded.

### Novel transcripts

A novel transcriptional active region (TAR) was determined in the intergenic regions by contiguous expression with each base supported by at least two uniquely mapped reads and a length > 35 bp. Supported by paired-end information, a novel transcript unit (TU) was constructed by connectivity between novel TARs that were joined by at least three paired reads. Novel TUs with length < 150 bp or average expression of < 2 reads per base were excluded from further analysis. Furthermore, the sequences of the novel transcripts were analyzed by ORF Finder in NCBI.

### UTR analysis

To define the UTRs of the annotated genes, we searched for a break in the transcribed region around the genes, denoted by positions with read counts of 0, starting from either end of genes. Genes where ends overlapped with other genes were excluded from the analysis. If no break was found, the UTR was discarded.

Upstream open reading frames (uORFs) and alternative upstream initiation codons (uATGs) were screened in the 5’-UTRs using computational methods. Any uORF of length > 150 bp or with a distance between the uORF and the gene start codon > 500 bp was excluded from the results. The length between the uATG and the gene’s coding region should be < 150 bp, uATGs must be in-frame with the gene’s coding region, and there should be no stop codon in the uATG sequence.

### **Analysis of IRES activity in 5’**-**UTR**

For IRES analysis, a set of bicistronic vectors was constructed (Table 2). The upstream cistron (*RML*) and the downstream cistron (β-galactosidase gene, *LacZ* from *E. coli* BL21) were cloned into the plasmid pGAPZαA (Invitrogen, USA), designated as the negative control plasmid, pRml-LacZ. Subsequently, the selected 5’-UTRs were amplified from *P. pastoris* cDNA and inserted between the two cistrons. The recombinant vectors were confirmed by DNA sequencing and transformed into *P. pastoris* X33. Together with the host *P. pastoris* X33, positive recombinant strains were cultivated on MDT plates (containing 5 g/L (NH_4_)_2_SO_4_, 3 g/L yeast extract, 1 g/L polyvinyl alcohol (PVA), 20 g/L agar, 5 mL/L tributyrin (Acros Organics, USA) and 100 mL/L 1 M phosphate buffer solution, pH 6.0) and MDX plates (containing 5 g/L (NH_4_)_2_SO_4_, 3 g/L yeast extract, 20 g/L agar, 4 mL/L X-gal solution (TaKaRa, Japan, 20 mg/mL buffered with dimethyl formamide) and 100 mL/L 1 M phosphate buffer solution, pH 6.0) to detect expression of the *RML* and β-galactosidase genes, respectively. To assess the integrity of the bicistronic mRNAs, PCR identification was performed with the corresponding cDNA as the template.

**Table 2:** Strains and plasmids constructed for IRES analysis

**Strains**	**Plasmids**	**Short descriptions**
X33/pRml-LacZ	pRml-LacZ	Negative control
X33/pRml-UTR-LacZ	pRml-UTR-LacZ	UTR was amplified from *KOG1* or *GCN2* gene
X33/pLacZ	pLacZ	Positive control of the expression of *LacZ*

### Northern blot analysis

*P. pastoris* strains X33/pRml-LacZ, X33/pRml-GCN2-LacZ, X33/pRml-KOG1-LacZ, and X33/pLacZ were cultivated in YPD (containing 1 g/L yeast extract, 2 g/L peptone, 2 g/L dextrose) for 48 h. Total RNA was isolated using the hot acidic phenol method and then denatured in a mixture of 50% formamide (Sigma), 17 mM morpholinepropane sulfonic acid (MOPS) and 2.2 M formaldehyde for 15 min at 65°C and chilled on ice. RNA was separated by electrophoresis on a 1% agarose gel containing 2.2 M formaldehyde and 20 mM MOPS and transferred onto a nylon membrane (Hybond-N, Amersham), on which they were cross-linked by UV. The preparation of the LacZ probe and subsequent hybridizations were performed using Express Hyb (Clontech) according to the manufacturer’s protocol.

### Gene ontology analysis

We obtained the GO terms of each *P. pastoris* gene with the Blast2GO software (ver. 2.4.9) [48] using default parameters. Blast2GO was also used for GO functional enrichment analysis of certain genes, performing Fisher’s exact test with a robust false discovery rate (FDR) correction to obtain an adjusted p-value between certain test gene groups and the whole annotation.

### Analysis of alternative splicing (AS) events

To identify all potential splice sites, we searched for the putative donor site (‘GT’ on the forward strand or ‘AC’ on the reverse strand) and the acceptor site (‘AG’ on the forward strand or ‘CT’ on the reverse strand) inside intronic regions and required that a novel splice site was to be supported by at least one uniquely mapped read. We then enumerated all the possible pairs of donor sites and acceptor sites to detect potential splice junctions.

To determine the number of reads supporting each splice junction, all reads that could not be matched to the genome and that could be matched by trimming off several terminal bases were retrieved and aligned against the junction sequence with a tolerance of 2 bp mismatches. A proposed junction site was required to be supported by at least two unambiguous mapped reads with different match positions within the splice junction region and also with a minimum match of five bases on each side of the junction (such reads were called “trans-reads”).

To identify AS events in *P. pastoris*, the junction sequences identified were used to analyze seven types of AS events including SE, RI, A5SS, A3SS, MXE, AFE and ALE according to the method of Wang [49].

### Analysis of differential gene expression under heterologous protein production conditions

Two biological replicates of SDEG and SDEM were used to analyze differentially expressed genes under culture conditions as in the description above, using the ‘R’ package (DEseq) [37], based on the read count of each gene under methanol or glycerol conditions. GO functional enrichment analysis was performed using the Blast2GO software and KEGG pathway analysis was performed using the Cytoscape software (ver. 2.6.2) [50].

### Validation of RNA-Seq analysis

Novel transcripts and new exons were validated by RT-PCR with primers designed in the exon regions (Additional file 2: Table S11). To detect different AS isoforms, real-time quantitative PCR (qPCR) was performed using primers designed inside exons or introns (Additional file 2: Table S11). The *P. pastoris* samples were the same as those for the Illumina sequencing. After extraction using the hot acidic phenol method, total RNA was treated with RNase-free DNase I (TaKaRa) for 45 min according to the manufacturer’s protocol. Subsequently, first-strand cDNA synthesis and qPCR were performed using the PrimeScript™ RT-PCR Kit (TaKaRa) and the SYBR Premix Ex Taq II™ kit (TaKaRa). For the negative control, the reverse transcriptase was omitted in the cDNA synthesis reaction with the DNase-treated total RNA as the template. No PCR product was obtained using the negative control as the template, suggesting that the genomic DNA was removed completed.

## Abbreviations

RNA-Seq: massively parallel mRNA sequencing; UTR: untranslated region; AS: alternative splicing; uATG: alternative upstream initiation codon; uORF: upstream open reading frame; IRES: internal ribosome entry site; RI: retained intron; ID: intron definition; TRAC: transcript analysis with the aid of affinity capture; RML: *Rhizomucor miehei* lipase; RPKM: reads per kilobase of exon region per million mapped reads; GO: gene ontology; FDR: false discovery rate; bp: base pair; LacZ: β-galactosidase; CE: cassette exon; SE: skipped exon; AFE: alternative first exon; ALE: alternative last exon; A5SS: alternative 5’-splice site; A3SS: alternative 3’-splice site; AOX: alcohol oxidase; FLD: formaldehyde dehydrogenase; FGH: S-formyl glutathione hydrolase; CAT: catalase; YFL054C: putative passive glycerol channel; STL1: active glycerol importer; GUT1: cytosolic glycerol kinase; RIN: RNA integrity number; TAR: transcriptional active region; TU: transcript unit; qPCR: real-time quantitative PCR.

## Competing interests

The authors declare that they have no competing interests.

## Authors’ contributions

SL prepared experiment samples, performed the experiment confirmation of transcriptome structure, participated in data analysis and manuscript revision. BW performed bioinfomatic data analysis and investigated the biological significance. MH mapped the sequencing reads to the reference genome. LP drafted the manuscript. YY contributed to the analysis of differential gene expression. SH, SZ contributed to help design the experiment. YL and XW designed the experiment, supervised the research and wrote the manuscript. All authors read and approved the final manuscript.

## Supplementary Material

Additional file 1 **Figure S1.** Description of experimental design and the samples. **Figure S2**. Overlapping transcripts in intergenic regions. **Figure S3**. GO functional enrichment analysis of *P. pastoris* gene expression of sample CKM. The blue cross band (“Test”) represents the percentage of genes belonging to each GO category in a certain gene group under CKM conditions, and the violet cross band (“Ref”) represents the percentage of genes belonging to each GO category in the reference group (GO annotated *P. pastoris* genes). The upper section is the gene group with an expression level higher than the 75^th^percentile, and the lower section is the gene group with an expression level lower than the 25^th^percentile. **Figure S4**. PCR experimental validation of novel transcripts and new exons. After removal of the genomic DNA by treating with DNase, total RNA was used to synthesize cDNA. (A) Validation of novel transcripts: 1 (there was no PCR product for amplifying actin using the negative control as templates), 2 (Actin, as the positive control), 3 (novel transcript in chromosome FN392323.1, coordinates 6187-6714), 4 (novel transcript in chromosome FN392320.1, coordinates 1550566-1550863), M, DNA molecular weight marker. (B) Validation of new exon. 1 (new exon in gene chr4_0246, coordinates 501191-501406), M, DNA molecular weight marker. **Figure S5**. Experimental validation of retained intron in chr1-1_0445. Chr1-3_0222, a transcribed gene without alternative splicing events, was used as a control. PCR primers were collected in Table S11. (A) Amplification plot of real time quantitative PCR of certain genes was as follows: 1, Actin; 2, exon of chr1-1_0445; 3, intron 2 of chr1-1_0445 (the retained intron); 4, exon of chr1-3_0222; 5, intron 1 of chr1-1_0445; 6, intron of chr1-3_0222. (B) Average Ct value of certain genes. **Figure S6**. KEGG metabolic pathway analysis of differentially expressed genes between SDEM and SDEG cultures. The network is represented by nodes linked with each other. Node size is enrichment significance of each term. Terms with up-regulated or down-regulated genes in the SDEM culture compared with SDEG culture are shown in red or green, and the color gradient represents the differential extent. Terms without differentially expressed genes are in white. Click here for file

Additional file 2 **Table S1.** Summary of sequencing and reads mapping. **Table S2**. Gene expression level. **Table S3**. Overlapping transcripts in intergenic regions. **Table S4**. UTR annotation in *P. pastoris* by RNA-Seq data. **Table S5**. uATGs in *P. pastoris*. **Table S6**. uORFs in *P. pastoris*. **Table S7**. Novel transcripts in *P. pastoris*. **Table S8**. New exons in *P. pastoris*. **Table S9**. AS events in *P. pastoris*. **Table S10**. Differentially expressed genes between *P. pastoris* SDEM and SDEG. **Table S11**. Primer list and the IRES sequences. Click here for file
