# Supplementary figures and images for: Comprehensive structural annotation of Pichia pastoris transcriptome and the response to various carbon sources using deep paired-end RNA sequencing
Source: BMC Genomics. 2012 Dec 31;13:738. doi: 10.1186/1471-2164-13-738 (PMC3547764; doi:10.1186/1471-2164-13-738)

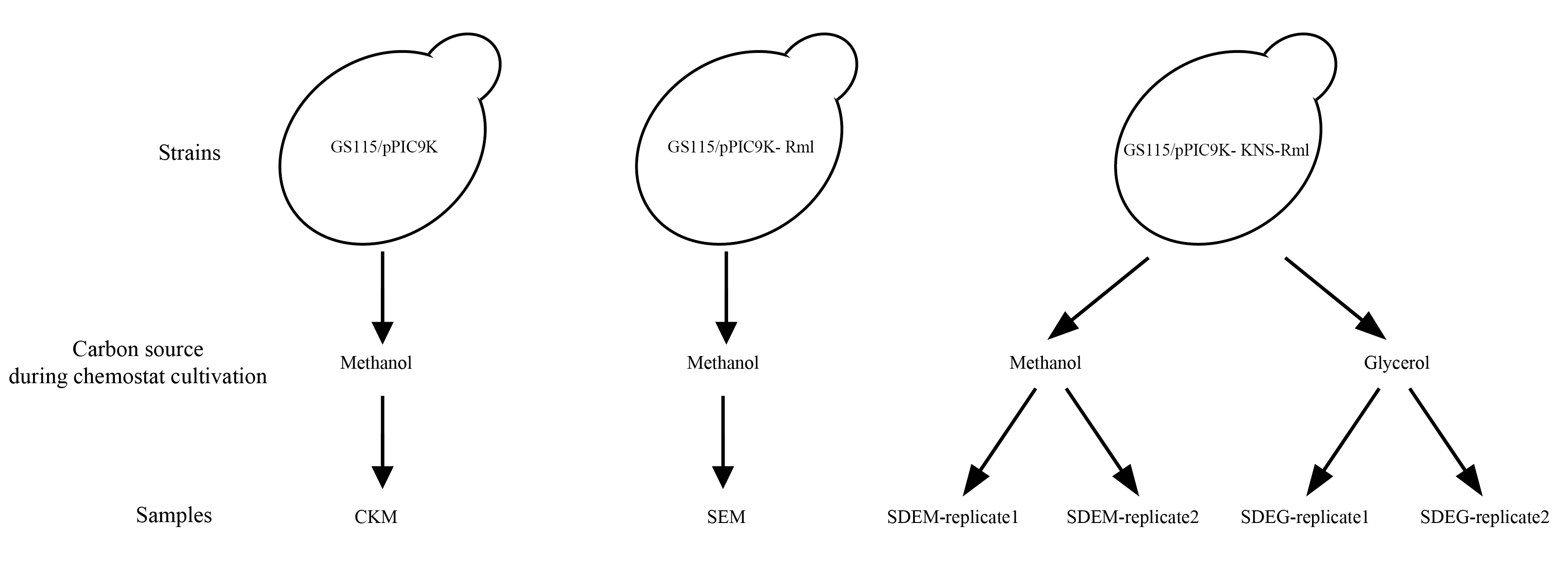

Supplement: Additional file 1 — Figure S1. Description of experimental design and the samples. Figure S2. Overlapping transcripts in intergenic regions. Figure S3. GO functional enrichment analysis of P. pastoris gene expression of sample CKM. The blue cross band (“Test”) represents the percentage of genes belonging to each GO category in a certain gene group under CKM conditions, and the violet cross band (“Ref”) represents the percentage of genes belonging to each GO category in the reference group (GO annotated P. pastoris genes). The upper section is the gene group with an expression level higher than the 75thpercentile, and the lower section is the gene group with an expression level lower than the 25thpercentile. Figure S4. PCR experimental validation of novel transcripts and new exons. After removal of the genomic DNA by treating with DNase, total RNA was used to synthesize cDNA. (A) Validation of novel transcripts: 1 (there was no PCR product for amplifying actin using the negative control as templates), 2 (Actin, as the positive control), 3 (novel transcript in chromosome FN392323.1, coordinates 6187-6714), 4 (novel transcript in chromosome FN392320.1, coordinates 1550566-1550863), M, DNA molecular weight marker. (B) Validation of new exon. 1 (new exon in gene chr4_0246, coordinates 501191-501406), M, DNA molecular weight marker. Figure S5. Experimental validation of retained intron in chr1-1_0445. Chr1-3_0222, a transcribed gene without alternative splicing events, was used as a control. PCR primers were collected in Table S11. (A) Amplification plot of real time quantitative PCR of certain genes was as follows: 1, Actin; 2, exon of chr1-1_0445; 3, intron 2 of chr1-1_0445 (the retained intron); 4, exon of chr1-3_0222; 5, intron 1 of chr1-1_0445; 6, intron of chr1-3_0222. (B) Average Ct value of certain genes. Figure S6. KEGG metabolic pathway analysis of differentially expressed genes between SDEM and SDEG cultures. The network is represented by nodes linked with each other. Node size is [file 1471-2164-13-738-S1.zip › 1008_1755971110663271_MOESM1_ESM/Additional file 1/Figure S1 (correction).png]

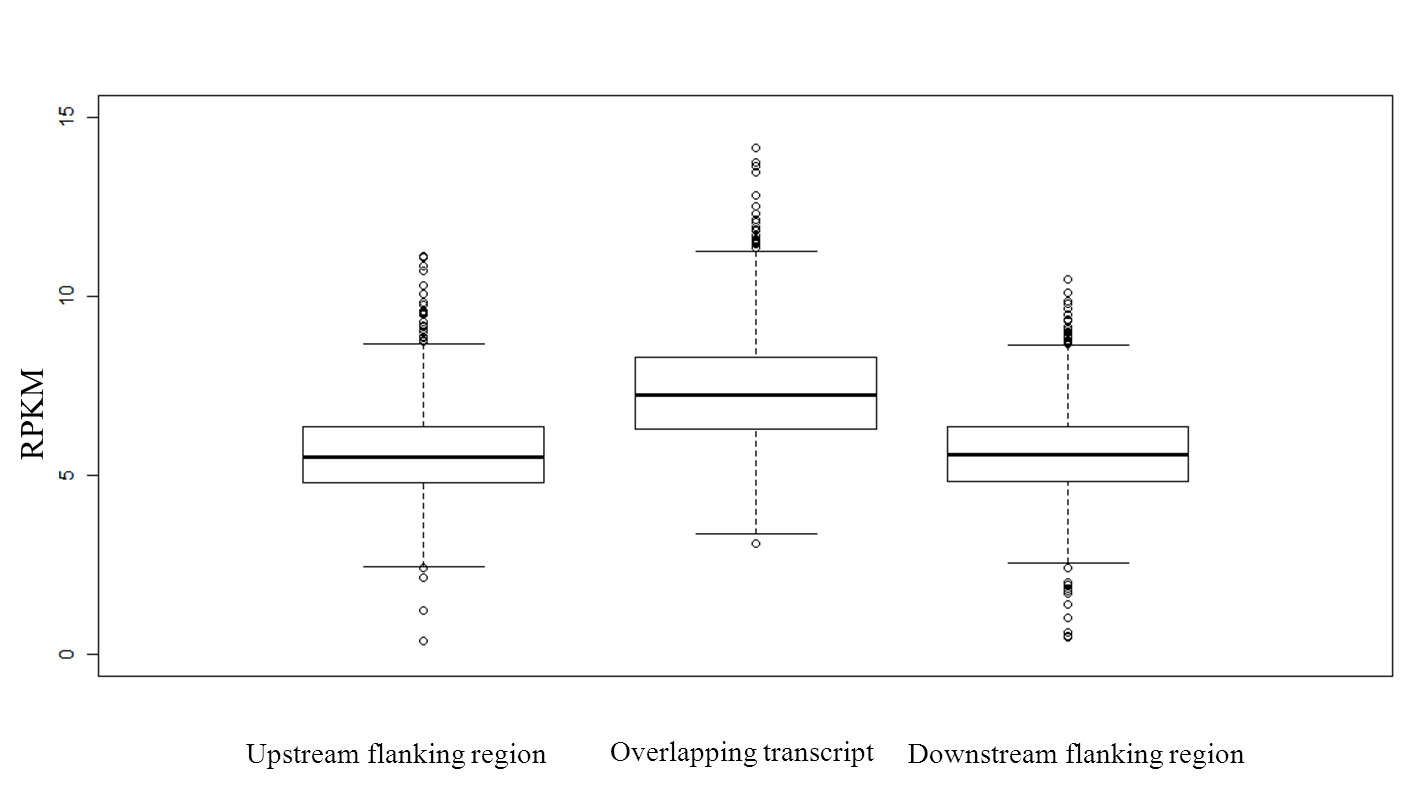

Supplement: Additional file 1 — Figure S1. Description of experimental design and the samples. Figure S2. Overlapping transcripts in intergenic regions. Figure S3. GO functional enrichment analysis of P. pastoris gene expression of sample CKM. The blue cross band (“Test”) represents the percentage of genes belonging to each GO category in a certain gene group under CKM conditions, and the violet cross band (“Ref”) represents the percentage of genes belonging to each GO category in the reference group (GO annotated P. pastoris genes). The upper section is the gene group with an expression level higher than the 75thpercentile, and the lower section is the gene group with an expression level lower than the 25thpercentile. Figure S4. PCR experimental validation of novel transcripts and new exons. After removal of the genomic DNA by treating with DNase, total RNA was used to synthesize cDNA. (A) Validation of novel transcripts: 1 (there was no PCR product for amplifying actin using the negative control as templates), 2 (Actin, as the positive control), 3 (novel transcript in chromosome FN392323.1, coordinates 6187-6714), 4 (novel transcript in chromosome FN392320.1, coordinates 1550566-1550863), M, DNA molecular weight marker. (B) Validation of new exon. 1 (new exon in gene chr4_0246, coordinates 501191-501406), M, DNA molecular weight marker. Figure S5. Experimental validation of retained intron in chr1-1_0445. Chr1-3_0222, a transcribed gene without alternative splicing events, was used as a control. PCR primers were collected in Table S11. (A) Amplification plot of real time quantitative PCR of certain genes was as follows: 1, Actin; 2, exon of chr1-1_0445; 3, intron 2 of chr1-1_0445 (the retained intron); 4, exon of chr1-3_0222; 5, intron 1 of chr1-1_0445; 6, intron of chr1-3_0222. (B) Average Ct value of certain genes. Figure S6. KEGG metabolic pathway analysis of differentially expressed genes between SDEM and SDEG cultures. The network is represented by nodes linked with each other. Node size is [file 1471-2164-13-738-S1.zip › 1008_1755971110663271_MOESM1_ESM/Additional file 1/Figure S2.png]

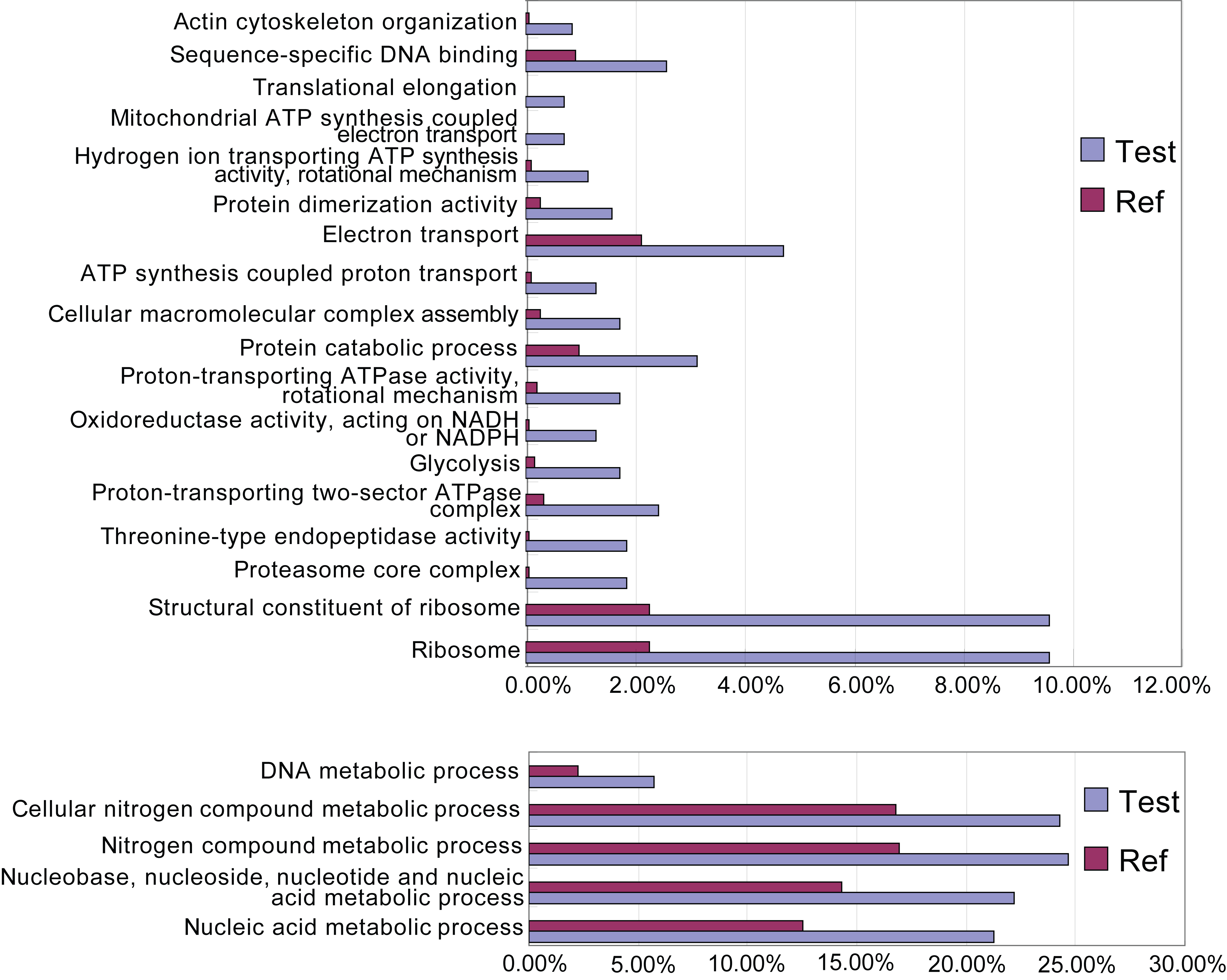

Supplement: Additional file 1 — Figure S1. Description of experimental design and the samples. Figure S2. Overlapping transcripts in intergenic regions. Figure S3. GO functional enrichment analysis of P. pastoris gene expression of sample CKM. The blue cross band (“Test”) represents the percentage of genes belonging to each GO category in a certain gene group under CKM conditions, and the violet cross band (“Ref”) represents the percentage of genes belonging to each GO category in the reference group (GO annotated P. pastoris genes). The upper section is the gene group with an expression level higher than the 75thpercentile, and the lower section is the gene group with an expression level lower than the 25thpercentile. Figure S4. PCR experimental validation of novel transcripts and new exons. After removal of the genomic DNA by treating with DNase, total RNA was used to synthesize cDNA. (A) Validation of novel transcripts: 1 (there was no PCR product for amplifying actin using the negative control as templates), 2 (Actin, as the positive control), 3 (novel transcript in chromosome FN392323.1, coordinates 6187-6714), 4 (novel transcript in chromosome FN392320.1, coordinates 1550566-1550863), M, DNA molecular weight marker. (B) Validation of new exon. 1 (new exon in gene chr4_0246, coordinates 501191-501406), M, DNA molecular weight marker. Figure S5. Experimental validation of retained intron in chr1-1_0445. Chr1-3_0222, a transcribed gene without alternative splicing events, was used as a control. PCR primers were collected in Table S11. (A) Amplification plot of real time quantitative PCR of certain genes was as follows: 1, Actin; 2, exon of chr1-1_0445; 3, intron 2 of chr1-1_0445 (the retained intron); 4, exon of chr1-3_0222; 5, intron 1 of chr1-1_0445; 6, intron of chr1-3_0222. (B) Average Ct value of certain genes. Figure S6. KEGG metabolic pathway analysis of differentially expressed genes between SDEM and SDEG cultures. The network is represented by nodes linked with each other. Node size is [file 1471-2164-13-738-S1.zip › 1008_1755971110663271_MOESM1_ESM/Additional file 1/Figure S3.png]

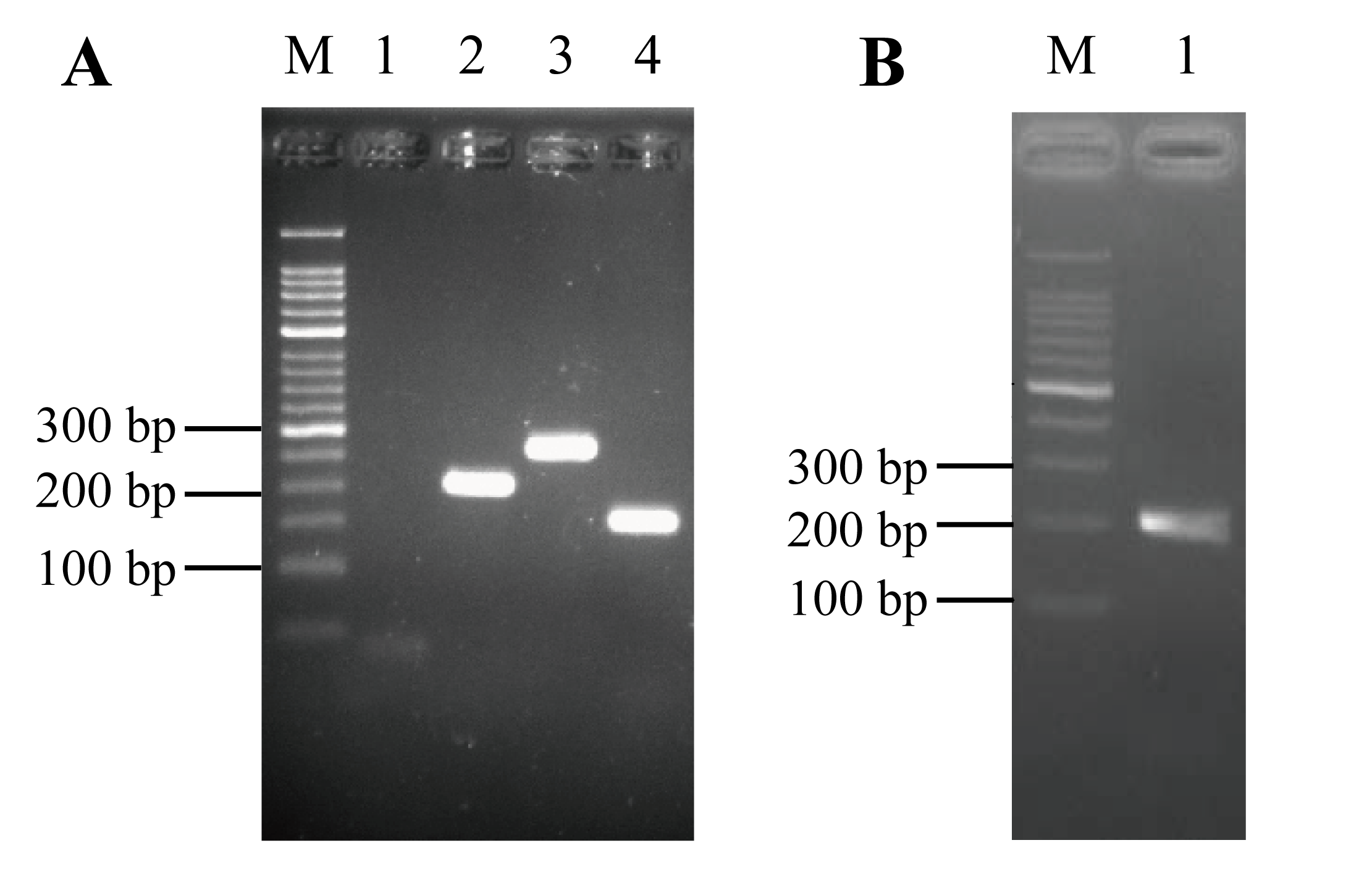

Supplement: Additional file 1 — Figure S1. Description of experimental design and the samples. Figure S2. Overlapping transcripts in intergenic regions. Figure S3. GO functional enrichment analysis of P. pastoris gene expression of sample CKM. The blue cross band (“Test”) represents the percentage of genes belonging to each GO category in a certain gene group under CKM conditions, and the violet cross band (“Ref”) represents the percentage of genes belonging to each GO category in the reference group (GO annotated P. pastoris genes). The upper section is the gene group with an expression level higher than the 75thpercentile, and the lower section is the gene group with an expression level lower than the 25thpercentile. Figure S4. PCR experimental validation of novel transcripts and new exons. After removal of the genomic DNA by treating with DNase, total RNA was used to synthesize cDNA. (A) Validation of novel transcripts: 1 (there was no PCR product for amplifying actin using the negative control as templates), 2 (Actin, as the positive control), 3 (novel transcript in chromosome FN392323.1, coordinates 6187-6714), 4 (novel transcript in chromosome FN392320.1, coordinates 1550566-1550863), M, DNA molecular weight marker. (B) Validation of new exon. 1 (new exon in gene chr4_0246, coordinates 501191-501406), M, DNA molecular weight marker. Figure S5. Experimental validation of retained intron in chr1-1_0445. Chr1-3_0222, a transcribed gene without alternative splicing events, was used as a control. PCR primers were collected in Table S11. (A) Amplification plot of real time quantitative PCR of certain genes was as follows: 1, Actin; 2, exon of chr1-1_0445; 3, intron 2 of chr1-1_0445 (the retained intron); 4, exon of chr1-3_0222; 5, intron 1 of chr1-1_0445; 6, intron of chr1-3_0222. (B) Average Ct value of certain genes. Figure S6. KEGG metabolic pathway analysis of differentially expressed genes between SDEM and SDEG cultures. The network is represented by nodes linked with each other. Node size is [file 1471-2164-13-738-S1.zip › 1008_1755971110663271_MOESM1_ESM/Additional file 1/Figure S4.png]

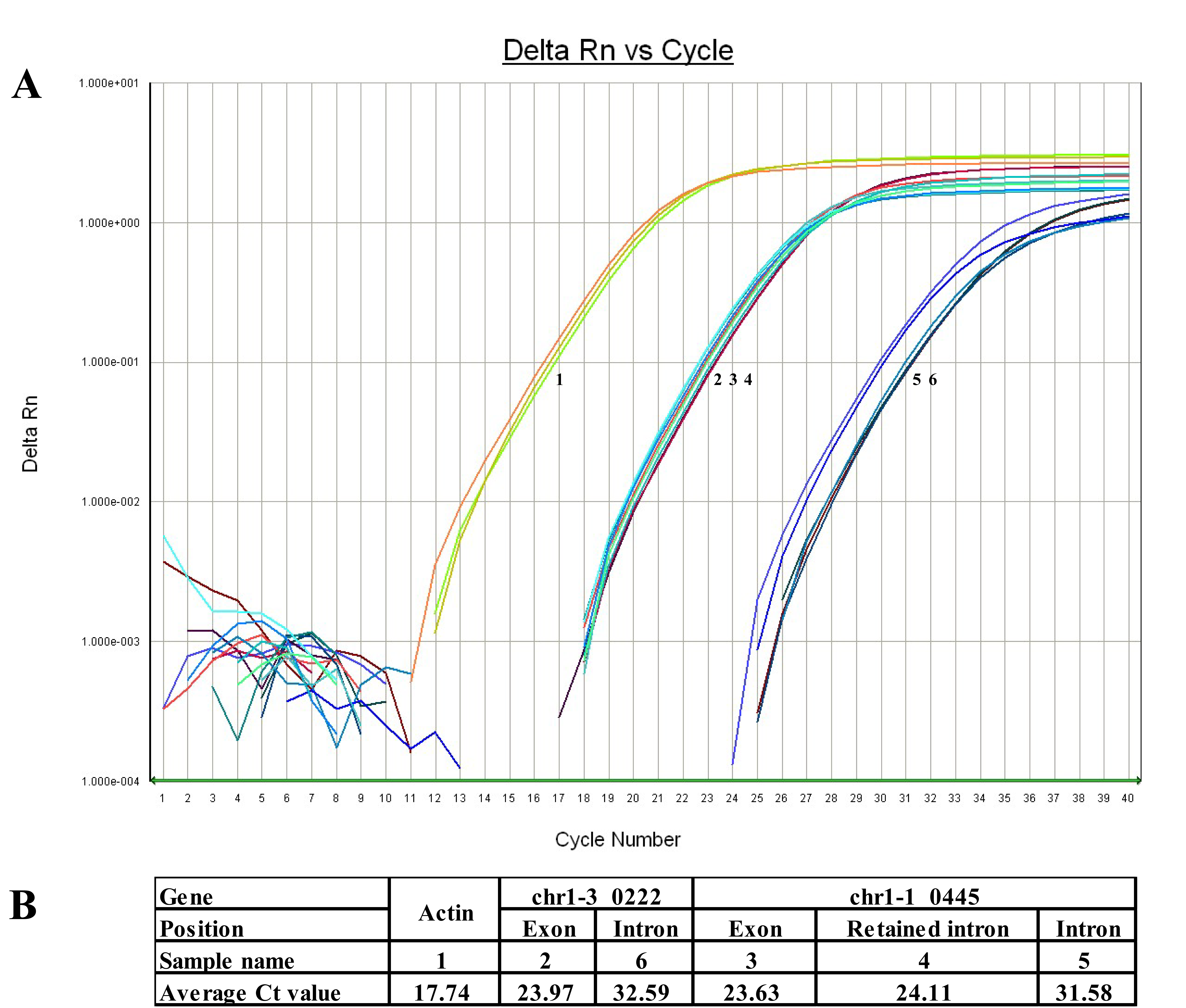

Supplement: Additional file 1 — Figure S1. Description of experimental design and the samples. Figure S2. Overlapping transcripts in intergenic regions. Figure S3. GO functional enrichment analysis of P. pastoris gene expression of sample CKM. The blue cross band (“Test”) represents the percentage of genes belonging to each GO category in a certain gene group under CKM conditions, and the violet cross band (“Ref”) represents the percentage of genes belonging to each GO category in the reference group (GO annotated P. pastoris genes). The upper section is the gene group with an expression level higher than the 75thpercentile, and the lower section is the gene group with an expression level lower than the 25thpercentile. Figure S4. PCR experimental validation of novel transcripts and new exons. After removal of the genomic DNA by treating with DNase, total RNA was used to synthesize cDNA. (A) Validation of novel transcripts: 1 (there was no PCR product for amplifying actin using the negative control as templates), 2 (Actin, as the positive control), 3 (novel transcript in chromosome FN392323.1, coordinates 6187-6714), 4 (novel transcript in chromosome FN392320.1, coordinates 1550566-1550863), M, DNA molecular weight marker. (B) Validation of new exon. 1 (new exon in gene chr4_0246, coordinates 501191-501406), M, DNA molecular weight marker. Figure S5. Experimental validation of retained intron in chr1-1_0445. Chr1-3_0222, a transcribed gene without alternative splicing events, was used as a control. PCR primers were collected in Table S11. (A) Amplification plot of real time quantitative PCR of certain genes was as follows: 1, Actin; 2, exon of chr1-1_0445; 3, intron 2 of chr1-1_0445 (the retained intron); 4, exon of chr1-3_0222; 5, intron 1 of chr1-1_0445; 6, intron of chr1-3_0222. (B) Average Ct value of certain genes. Figure S6. KEGG metabolic pathway analysis of differentially expressed genes between SDEM and SDEG cultures. The network is represented by nodes linked with each other. Node size is [file 1471-2164-13-738-S1.zip › 1008_1755971110663271_MOESM1_ESM/Additional file 1/Figure S5.png]

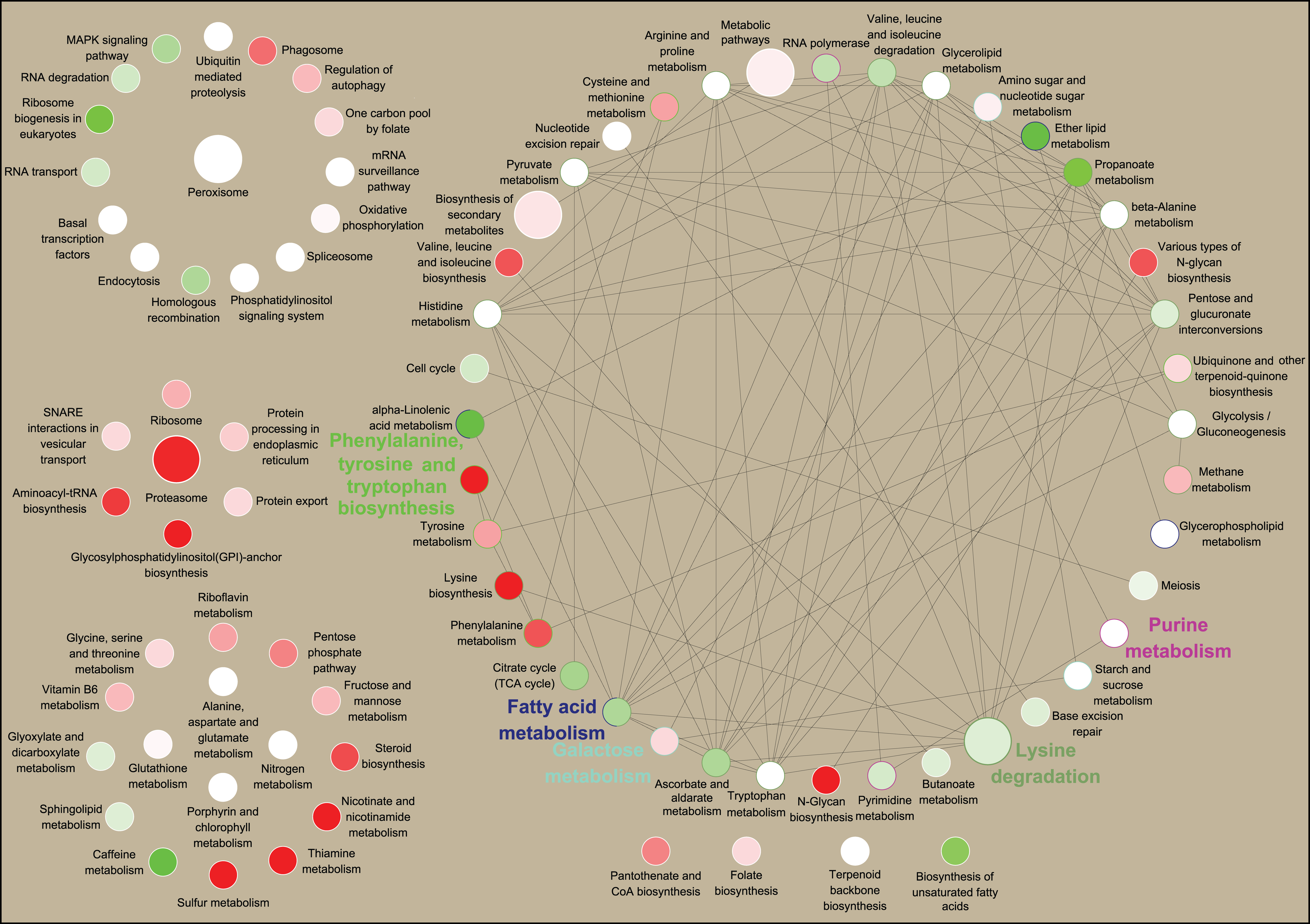

Supplement: Additional file 1 — Figure S1. Description of experimental design and the samples. Figure S2. Overlapping transcripts in intergenic regions. Figure S3. GO functional enrichment analysis of P. pastoris gene expression of sample CKM. The blue cross band (“Test”) represents the percentage of genes belonging to each GO category in a certain gene group under CKM conditions, and the violet cross band (“Ref”) represents the percentage of genes belonging to each GO category in the reference group (GO annotated P. pastoris genes). The upper section is the gene group with an expression level higher than the 75thpercentile, and the lower section is the gene group with an expression level lower than the 25thpercentile. Figure S4. PCR experimental validation of novel transcripts and new exons. After removal of the genomic DNA by treating with DNase, total RNA was used to synthesize cDNA. (A) Validation of novel transcripts: 1 (there was no PCR product for amplifying actin using the negative control as templates), 2 (Actin, as the positive control), 3 (novel transcript in chromosome FN392323.1, coordinates 6187-6714), 4 (novel transcript in chromosome FN392320.1, coordinates 1550566-1550863), M, DNA molecular weight marker. (B) Validation of new exon. 1 (new exon in gene chr4_0246, coordinates 501191-501406), M, DNA molecular weight marker. Figure S5. Experimental validation of retained intron in chr1-1_0445. Chr1-3_0222, a transcribed gene without alternative splicing events, was used as a control. PCR primers were collected in Table S11. (A) Amplification plot of real time quantitative PCR of certain genes was as follows: 1, Actin; 2, exon of chr1-1_0445; 3, intron 2 of chr1-1_0445 (the retained intron); 4, exon of chr1-3_0222; 5, intron 1 of chr1-1_0445; 6, intron of chr1-3_0222. (B) Average Ct value of certain genes. Figure S6. KEGG metabolic pathway analysis of differentially expressed genes between SDEM and SDEG cultures. The network is represented by nodes linked with each other. Node size is [file 1471-2164-13-738-S1.zip › 1008_1755971110663271_MOESM1_ESM/Additional file 1/Figure S6.png]
